# Early and Midterm Outcomes of Atrial Myxoma Resection Via Superior Transseptal Approach

**DOI:** 10.5152/eurasianjmed.2026.251151

**Published:** 2026-01-15

**Authors:** Hande İştar, Buğra Harmandar, Melike Korkmaz Toker

**Affiliations:** 1 Department of Cardiovascular Surgery, Muğla Sıtkı Koçman University Faculty of Medicine, Muğla, Türkiye; 2 Department of Anaesthesiology & Reanimation, Muğla Sıtkı Koçman University Faculty of Medicine, Muğla, Türkiye

**Keywords:** Arrhythmia, atrium, myxoma, recurrence, tumor

## Abstract

**Background:**

Atrial myxomas are the most common primary cardiac neoplasms, typically presenting with obstructive cardiac symptoms or embolic events. Surgical excision remains the definitive treatment; however, concerns persist regarding surgical exposure, postoperative arrhythmia, and recurrence. This study compared the early and mid-term outcomes of atrial myxoma excision performed exclusively using the superior transseptal technique with outcomes reported in the current literature.

**Methods:**

This retrospective cohort study included 10 patients who underwent surgical resection of atrial myxoma using a superior transseptal approach between January 2018 and December 2025. Demographic, preoperative, intraoperative, and postoperative data were collected. Outcomes analyzed included perioperative morbidity and mortality, rhythm disturbances, recurrence, and length of hospital stay.

**Results:**

The cohort consisted of 7 women and 3 men with a mean age of 52.9 ± 9.0 years. Dyspnea was the most common presenting symptom (70%), and embolic events occurred in 10% of patients. All tumors were excised en bloc with a surrounding margin of septal tissue; 100% required atrial septal defect closure using an autologous pericardial patch. Postoperative atrial fibrillation was observed in 50% of patients, though none required permanent pacemaker implantation. There was no in-hospital mortality or tumor recurrence during a mean follow-up period of 3.5 ± 1.6 years.

**Conclusion:**

The superior transseptal approach provided optimal exposure and enabled safe, effective resection of atrial myxomas, resulting in no mortality, no recurrence, and acceptable complication rates. These findings support the use of this technique as a reliable strategy in the surgical management of atrial myxomas.

**Main Points**

- The superior transseptal technique is safe for myxoma resection and provides better exposure.
- With the superior transseptal technique, no operative mortality, no recurrence occurred.
- Mixoma operation using the superior transseptal technique provides a favorable early and midterm outcome.

## Introduction

Primary cardiac myxomas remain the most prevalent benign cardiac tumors, with an annual incidence of approximately 0.5-0.7 per million individuals.^1^

Myxomas are benign cardiac lesions that can be resolved through surgical intervention. They originate from primitive, undifferentiated mesenchymal cells and may be sessile or pedunculated. Typically located in the intracavitary space, they can exhibit local invasion. Although they can occur in any chamber of the heart, they are most frequently found in the left atrium, accounting for 75% of cases.^2^ They may also be present in both atria, and in rare cases—even in infants- have been reported to arise from the right atrial appendage.^3^^,^^4^

These tumors most often present in women in their fifth decade of life and typically originate in the left atrium. Larger tumors are associated with symptoms such as dyspnea, orthopnea, paroxysmal nocturnal dyspnea, pulmonary edema, wheezing, and hemoptysis.^5^ Although myxomas are benign, they can occasionally lead to sudden death due to protrusion into the left ventricle through the mitral valve.

Classic clinical manifestations include systemic embolism, fatigue, fever, and weight loss, often accompanied by arrhythmias and heart failure.^5^^,^^6^ Myxomas may also signal the presence of Carney complex, an autosomal dominant syndrome characterized by a spectrum of clinical features, including endocrinopathy, endocrine and non-endocrine tumors, and abnormal skin pigmentation.^5^

Recent meta-analyses have confirmed that surgical resection remains the standard treatment, with a median early mortality rate below 2%, a recurrence rate near 2%, and long-term survival rates ranging from 75% to 100% over a span of up to 30 years.^7^

Despite generally favorable outcomes, postoperative atrial fibrillation (AF) is a common complication (>10%), and embolic events may recur even in the early postoperative period, particularly in patients who presented with preoperative cerebrovascular events. Minimally invasive techniques for left atrial myxoma resection are gaining traction, demonstrating safety and efficacy comparable to conventional median sternotomy, though confirmation in larger cohorts is still pending.^8^

The aim of this study was to assess large atrial myxomas operated on using a superior transseptal incision and to enhance understanding of atrial myxoma treatment by integrating empirical data within the context of advanced diagnostic methods and surgical techniques. The series of 10 patients provides a current dataset reflecting recent developments in perioperative imaging, surgical approaches, and innovative care regimens. The objectives were to identify the demographic and clinicopathologic characteristics of the cohort and to evaluate early and midterm surgical outcomes, including mortality, arrhythmia, and recurrence.

## Material and Methods

### Study Design

From January 2018 through June 2025, data were analyzed from individuals who underwent surgical resection of atrial myxoma at the hospital. This retrospective cross-sectional study was conducted in accordance with the principles outlined in the Declaration of Helsinki and received Ethics Committee approval from Muğla Sıtkı Koçman University Medical Faculty institutional review board (approval no: (date: August 07, 2025-Approval no: 250170-154). Informed consent was obtained from all participants. The study aimed to evaluate early and midterm surgical outcomes, including mortality, arrhythmia, recurrence; and to identify related risk factors.

### Patient Selection

Patients over 18 years of age with histopathologically confirmed atrial myxoma who underwent surgical removal were included. The exclusion criteria were incomplete clinical data and the presence of non-myxomatous cardiac tumors.

### Data Collection

Data were collected from hospital records and electronic medical records. Preoperative variables included demographics (age, sex); follow-up duration; history of myxoma recurrence; familial myxoma history; presence of Carney complex (a rare autosomal dominant genetic disorder associated with multiple endocrine neoplasia syndromes or with primary pigmented nodular adrenal disease. It affects various endocrine glands such as the thyroid, pituitary, and adrenal glands); concomitant cardiac conditions; preoperative rhythm; ejection fraction (EF); involved cardiac chamber; and the occurrence of embolic events, cerebrovascular incidents, heart failure, syncope, and urgent presentation.

Intraoperative data included cardiopulmonary bypass (CPB) duration, aortic cross-clamp (ACC) time, tumor location, tumor morphology (pedunculated vs. sessile), tumor size, and any concomitant procedures (e.g., atrial septal defect (ASD) repair, tricuspid valve repair, left atrial roof patch repair).

Postoperative variables encompassed revision surgery for bleeding, duration of mechanical ventilation, length of stay in the intensive care unit (ICU), total hospital length of stay, postoperative arrhythmias, need for permanent pacemaker implantation, cerebrovascular events, low cardiac output syndrome, acute kidney injury, persistent pericardial effusion, and mortality.

### Surgical Technique

All surgeries were performed under general anesthesia via median sternotomy. After initiating CPB through bicaval cannulation and administering Del Nido cardioplegia, diastolic cardiac arrest was achieved. All patients underwent tumor resection using a superior transseptal incision to allow optimal exposure of the tumor and adjacent structures. The tumor was excised along with a substantial portion of the interatrial septum. In all cases, the ASD was repaired with an autologous pericardial patch. When indicated, additional procedures—such as tricuspid valve repair or left atrial roof patch repair—were performed concurrently.

### Statistical Analysis

All statistical analyses were conducted using IBM SPSS Statistics version 21.0 (IBM Corp., Armonk, NY, USA). Continuous variables are presented as mean ± standard deviation, while categorical variables are reported as counts and percentages.

## Results

Ten patients underwent surgical resection of atrial myxoma via the superior transseptal approach at the institution between 2018 and 2025. The majority were female (70%), with a mean age of 52.9 years (range: 35-65 years). Over a mean follow-up period of 3.5 years, no perioperative mortality or tumor recurrence was observed. Demographic characteristics—including age distribution, sex, follow-up duration, and genetic or familial features—are summarized in Table 1.

Sixty percent of patients presented with sinus rhythm prior to surgery. The mean preoperative EF was 59.0% ± 3.9%. Dyspnea was the most common symptom, reported in 70% of cases, followed by palpitations in 50%. Cerebrovascular events and peripheral embolism were each observed in 10% of patients. Additional valvular heart diseases were present in some cases, with aortic insufficiency observed in 30% and mitral insufficiency in 10% of patients (Table 2).

Intraoperative details are summarized in Table 3. The mean CPB time was 75.0 ± 10.0 minutes, and the mean ACC time was 45.0 ± 8.0 minutes. Most tumors (80%) were located in the left atrium, with 50% displaying a pedunculated morphology. The average maximal tumor diameter was 6 cm. Atrial septal defect repair was performed in all cases. One patient required concurrent tricuspid repair, while another underwent left atrial roof patch repair.

No patient required revision surgery for bleeding. The mean duration of mechanical ventilation was 5.5 ± 1.1 hours. The mean ICU stay was 3.1 ± 0.9 days, and the mean hospital length of stay was 12.9 ± 4.9 days. Postoperatively, sinus rhythm was restored or maintained in 50% of patients, while the remaining 50% developed AF. No cases of complete heart block, permanent pacemaker implantation, cerebrovascular events, low cardiac output syndrome, or in-hospital mortality were observed. Acute kidney injury occurred in 1 patient (10%), and prolonged pericardial effusion was noted in 7 patients (70%) (Table 4).

## Discussion

In this retrospective cohort of 10 patients who underwent surgical resection of atrial myxoma using the superior transseptal approach, it was observed that exemplary early and mid-term outcomes, including no mortality, no recurrence, and acceptable perioperative complication rates. The prevalence of left atrial myxomas (80%) and female patients (70%) in the sample aligns with historical data, particularly the series documented by Tansel et al (2006).^3^

Dyspnea was the predominant presenting symptom (70%), reflecting the hemodynamic effects of left atrial tumors impeding mitral inflow. The incidence of embolic events in the cohort was 10%, which is lower than the 30%-40% reported in previous studies such as Tansel et al,^3^ suggesting a potential role for earlier diagnosis and intervention facilitated by contemporary imaging modalities.

Postoperative AF occurred in 50% of the patients, consistent with rates reported in larger cohorts and meta-analyses, underscoring the arrhythmogenic potential of atriotomy regardless of the technique used. Notably, no patients required permanent pacemaker implantation, indicating the precision of the surgical technique and the effectiveness of careful atrial repair.

Prolonged pericardial effusion, observed in 70% of patients, was asymptomatic and managed conservatively in all cases. This finding underscores the importance of diligent postoperative monitoring.

The primary objective of surgery is complete tumor excision. Manipulation of the heart prior to achieving cardiac arrest is strongly discouraged to prevent systemic embolization. To optimize visualization of the tumor and cardiac valves, and to assess for multicentricity, several incisions have been proposed, including the biatrial transseptal approach (Dubost incision), the superior transseptal approach, and left atriotomy via the Waterstone–Sundergard groove. These techniques recommend excising as much of the atrial septum as possible around the tumor base. ^9^^,^^10^

In redo operations, cases with a small left atrial cavity or large friable tumors in the left atrium, it is required an enhanced exposure. Although the biatrial technique offers excellent visualization via 2 atrial incisions, it has been criticized for its high rate of postoperative rhythm disturbances.^11^ According to Sellke et al,^11^ this method is associated with a 12% recurrence reduction. However, they also reported that 12% of patients developed complete heart block and 46% experienced AF or atrial flutter. In this series, the superior transseptal approach was preferred for better exposure, particularly in the case of a small-sized left atrium. Moreover, myxomas grow rapidly; therefore, the left atrial cavity does not expand as much as in mitral insufficiency or mitral stenosis.

The superior transseptal technique used in all cases provided excellent visibility for thorough tumor resection. Current literature emphasizes the value of this approach in achieving clear resection margins, especially when exposure of the left atrium is challenging.^12^^,^^13^ While some studies have raised concerns about prolonged CPB and ACC times associated with this incision, the surgical durations in our series remained within acceptable limits and had no adverse effect on outcomes.^14^ We argued that, minimizing the tumor recurrence by preventing tumor seeding within the cavity and protecting adjacent structures such as the mitral valve, is equally important as the precision of the resection itself.

Atrial myxomas typically originate from the limbus of the fossa ovalis. Recurrence after resection is an undesirable outcome that may result from incomplete resection of the septal wall, intraoperative tumor seeding, or the tumor’s multicentric nature. No recurrences were observed in the current series.

 Our findings support growing evidence that the superior transseptal approach allows for safe and effective resection of atrial myxomas, providing excellent visualization with acceptable complication rates, consistent with the results reported by Emiroğulları et al^15^ and Tenpaku et al.^16^

Yüksel et al’s^17^ study including 39 patients diagnosed with atrial myxoma evaluated postoperative outcomes in regard to different atrial incisions.They used biatrial incisions in 79.1% of their cohort and concluded that biatrial incisions are beneficial for valvular procedures and myxoma excision in the same session. Also, Çetintaş et al’s^18^ study revealed that valvular and myxoma resection can be operated in the same session with biatrial septal incision safely.

Median sternotomy is the most preferred approach. Mendyka et al^19^ compared median sternotomy, minimally invasive approach, and robotic-assisted approach. They concluded that the minimally invasive method and robotic-assisted approach provide lower postoperative blood loss, lower transfusion rates, shorter postoperative periods, faster recovery, and lower incidence of arrhythmia rate.^19^

Further multicenter studies incorporating modern surgical techniques and updated perioperative care protocols are needed to refine strategies for preventing arrhythmias and optimizing patient outcomes following atrial myxoma excision.

This series demonstrates the safe surgical removal of atrial myxomas using the superior transseptal technique, resulting in no operative mortality, no recurrence, and favorable early and mid-term outcomes. The approach provided optimal exposure, enabling complete tumor excision while preserving functional integrity. Our findings suggest that this method should be considered a reliable and effective option in the surgical management of atrial myxomas.

## Figures and Tables

**Table 1.** Demographic Characteristics of Patients

Characteristic	n
Sex (male/female)	3/7 (30/70)
Age (years)	52.9 ± 9.0
Follow-up duration (years)	3.5 ± 1.6
Myxoma recurrence	0 (0)
Familial myxoma	0 (0)
Carney complex	1 (10)

Data are presented as n (%) or mean ± standard deviation. n: number.

**Table 2.** Preoperative Characteristics of Patients

Characteristics	n
Preoperative sinus rhythm	6 (60)
Preoperative EF (%)	59.0 ± 3.9
Dyspnea	7 (70)
Palpitation	5 (50)
Peripheral embolism	1 (10)
Cerebrovascular event	1 (10)
Pulmonary embolism	0 (0)
Concomitant cardiac pathology CAD Congestive heart failure Mitral insufficiency Aortic insufficiency	0 (0) 0 (0) 3 (30) 1 (10)
Pericardial effusion	2 (20)
Syncope	2 (20)
Urgent operation	0 (0)

Data are presented as n (%) or mean ± standard deviation.

CAD, coronary artery disease; EF, ejection fraction.,n: number.

**Table 3.** Intraoperative Variables

Variables	n
CPB time (minutes)	75.0 ± 10.0
ACC time (minutes)	45.0 ± 8.0
LA myxoma	8 (80)
RA myxoma	2 (20)
Peduncle	5 (50)
Maximum tumor diameter (cm)	6.0 ± 0.9
ASD repair	10 (100)
Tricuspid valve repair	1 (10)
LA roof patch repair	1 (10)

Data are presented as n (%) or mean ± standard deviation.

ACC, aortic cross-clamp; ASD, atrial septal defect; CPB, cardiopulmonary bypass; LA, left atrial; RA, right atrial., n:number.

**Table 4.** Postoperative Variables

Variables	n
Revision for bleeding	0 (0)
Duration of mechanical ventilatory support (hours)	5.5 ± 1.1
ICU stay (days)	3.1 ± 0.9
In-hospital stay (days)	12.9 ± 4.9
Postoperative rhythm Sinus AF Block	5 (50) 5 (50) 0 (0)
Permanent pacemaker	0 (0)
Cerebrovascular event	0 (0)
Low CO	0 (0)
AKI	1 (10)
Prolonged pericardial effusion	7 (70)
Mortality	0 (0)

Data are presented as n (%) or mean ± standard deviation.

AF, atrial fibrillation; AKI, acute kidney injury; CO, cardiac output; ICU, intensive care unit.n: number.

## Data Availability

The datasets generated during and/or analyzed during the current study are available from the corresponding author on reasonable request.
